# Mitogenomic analyses support the recent division of the genus *Orthotrichum* (Orthotrichaceae, Bryophyta)

**DOI:** 10.1038/s41598-017-04833-z

**Published:** 2017-06-30

**Authors:** Jakub Sawicki, Vítězslav Plášek, Ryszard Ochyra, Monika Szczecińska, Monika Ślipiko, Kamil Myszczyński, Tomasz Kulik

**Affiliations:** 1 Department of Botany and Nature Protection, University of Warmia and Mazury, Plac Łódzki 1, 10-727 Olsztyn, Poland; 2 Department of Biology and Ecology, University of Ostrava, Chittussiho 10, 710 00 Ostrava, Czech Republic; 3 Laboratory of Bryology, Institute of Botany, Polish Academy of Sciences, Lubicz 46, 31-512 Kraków, Poland

## Abstract

A recently presented taxonomical arrangement of the moss genus *Orthotrichum* Hedw. s.l. substantially changed the traditional view of the taxon that had been accepted throughout the twentieth century. This paper provides the results of mitogenomic studies that strongly support the new taxonomical concept. Comparative analyses presented in this study confirmed the stable structure of moss mitogenomes. Moreover, 17 complete mitogenome sequences were used to identify the major evolutionary groups, including 11 newly sequenced ones, for this study. The analysis of mitochondrial hotspots revealed intron 4 of the *cox*1 gene to be the most variable non-coding region. The most variable protein-coding genes in the tribe Orthotricheae were *ccm*FC and *tat*C. The intergenic and intronic hotspots of *Orthotrichum* s.l. identified in the present study do not correspond to those described in vascular plant mitogenomes.

## Introduction

The genera *Nyholmiella* Holmen & Warncke, *Lewinskya* F. Lara, Garilleti & Goffinet and *Orthotrichum* Hedw. represent a group of approximately 167 species widely distributed subcosmopolitan mosses of the family Orthotrichaceae^1–13^. Species of these genera are found in several biomes, with the exception of deserts and wet tropical forests. *Orthotrichum* species are predominantly epiphytes that grow on tree trunks and branches, and they are less often found in saxicolous habitats at high altitudes, up to 5,000 m a.s.l.^14^. The subdivision within this genus has been the subject of continuing debate since the late 19th century. Specific taxa have been repeatedly included in and removed from *Orthotrichum* s.l. in an attempt to identify infrageneric taxa, including subgenera and sections. The history of *Orthotrichum* s.l. taxonomic classification was described in detail by Lewinsky and Hedenäs^14, 15^, and several phylogenetic analyses revealed that the genus was polyphyletic^16–19^. Nuclear and chloroplastic molecular markers were used to identify four groups within *Orthotrichum* s.l. with variable reproductive systems and localization of stomata^17, 18^. Based on these molecular results, a new taxonomical arrangement was proposed for the group, in which *Orthotrichum* s.l. was divided into the genera *Lewinskya*, *Nyholmiella*, *Orthotrichum* and *Pulvigera* Plášek, Sawicki & Ochyra^20, 21^.

Moreover, previous research demonstrated that the *Orthotrichum* s.l. evolutionary and taxonomic debate would not be resolved without the inclusion of species from the genus *Ulota* Mohr., which is closely related to the genus *Lewinskya* ^16, 17, 19^. *Ulota* contains approximately 60 species^22^, more than half of which are found in the southern hemisphere. The genus is characterized by superficial stomata, highly differentiated basal leaf cells, crisped leaves, and a lack of brood bodies on the leaf lamina. Based on results of molecular studies^17, 19^, the dioecious *U*. *phyllantha* Brid. was transferred to the separate monospecific genus *Plenogemma* Plášek, Sawicki & Ochyra^20^. The genera mentioned above, together with *Sehnemobryum* Lewinsky-Haapasaari & Hedenäs and *Stoneobryum* D.H. Norris & H. Rob., are classified in the tribe Orthotricheae^17^.

The genera of the tribe Orthotricheae comprise monoecious (*Lewinskya*, *Orthotrichum* s.s., *Sehnemobryum*, *Ulota*) and dioecious (*Nyholmiella*, *Plenogemma*, *Pulvigera* and *Stoneobryum*) taxa, which raises questions about the ancestry and monophyly of taxa representing the different sexual systems^14^. The tribe Zygodonteae, sister to Orthotricheae, is characterized by dioecy, which suggests dioecy as the ancestral state of Orthotricheae. The sexual system in Orthotricheae is usually correlated with the position of stomata; dioecious taxa are characterized by phaneroporous stomata (*Nyholmiella*, *Plenogemma*, and *Pulvigera*), except S*toneobryum*, which is the only known dioecious genus with cryptoporous stomata. Remaining cryptoporous species are monoecious and are classified into the monophyletic genus *Orthotrichum* s.s. The phylogenetic position of *Stoneobryum* will determine whether the transformation from phaneroporous to cryptoporous stomata independently appeared only once or twice during the evolution of the tribe.

Molecular markers and phylogenetic analyses have been effectively utilized to distinguish between the genera of the tribe Orthotricheae, and these relationships have been supported with high bootstrap values and significant Bayesian posterior probabilities^17, 18, 23^. However, despite the strong statistical support, data poorly support the determination of mutual phylogenetic relationships, because phylogenetic trees often differ depending on the sequences analyzed.

Next-generation sequencing supports phylogenetic reconstruction based on the complete sequences of organellar genomes^24, 25^. In animals, complete mitochondrial genomes containing tens of thousands of base pairs are commonly used in phylogenetic reconstruction^26^. The mitochondrial genomes of vascular plants are characterized by highly diverse structures, but these genomes are rather conservative in mosses^27^. However, plant phylogenetic analyses mostly rely on plastid gene sequences, which are characterized by a relatively stable structure and size at lower taxonomic levels and a higher rate of evolution that increases the number of parsimony informative mutations^28^. Moreover, the absence of structural changes in the mitochondrial genomes of mosses facilitates the analysis of these genomes and their phylogenetic reconstruction. To date, mitochondrial genomes have rarely been used to investigate the evolution of lower taxonomic plant groups. Although mitochondrial genomes (hereafter referred to as ‘mitogenomes’) evolve more slowly than plastomes and nuclear genomes, they may be useful for the resolution of phylogenetic relationships in some cases where traditional methods fail. For instance, studies on the broadly understood genus *Racomitrium* Brid. commonly use nuclear ITS regions that are too variable for intergeneric studies, resulting in controversial alignments^29^. Furthermore, commonly used plastid regions (*trn*H-*psb*A, *mat*K) have revealed a rather small number of parsimony informative sites^29, 30^. However, recent *Racomitrium* studies that used complete mitogenomes supported splitting this genus into five distinct clades^27^.

The main goal of the current study was to provide molecular support for the recent taxonomical rearrangements of the tribe Orthotricheae^17, 19, 20^ and to evaluate the usefulness of mitogenomes for evolutionary research at the genus level. Comparative genomics will provide information about mitogenomic hotspots for further phylogenetic studies.

## Results

**Figure 1:**
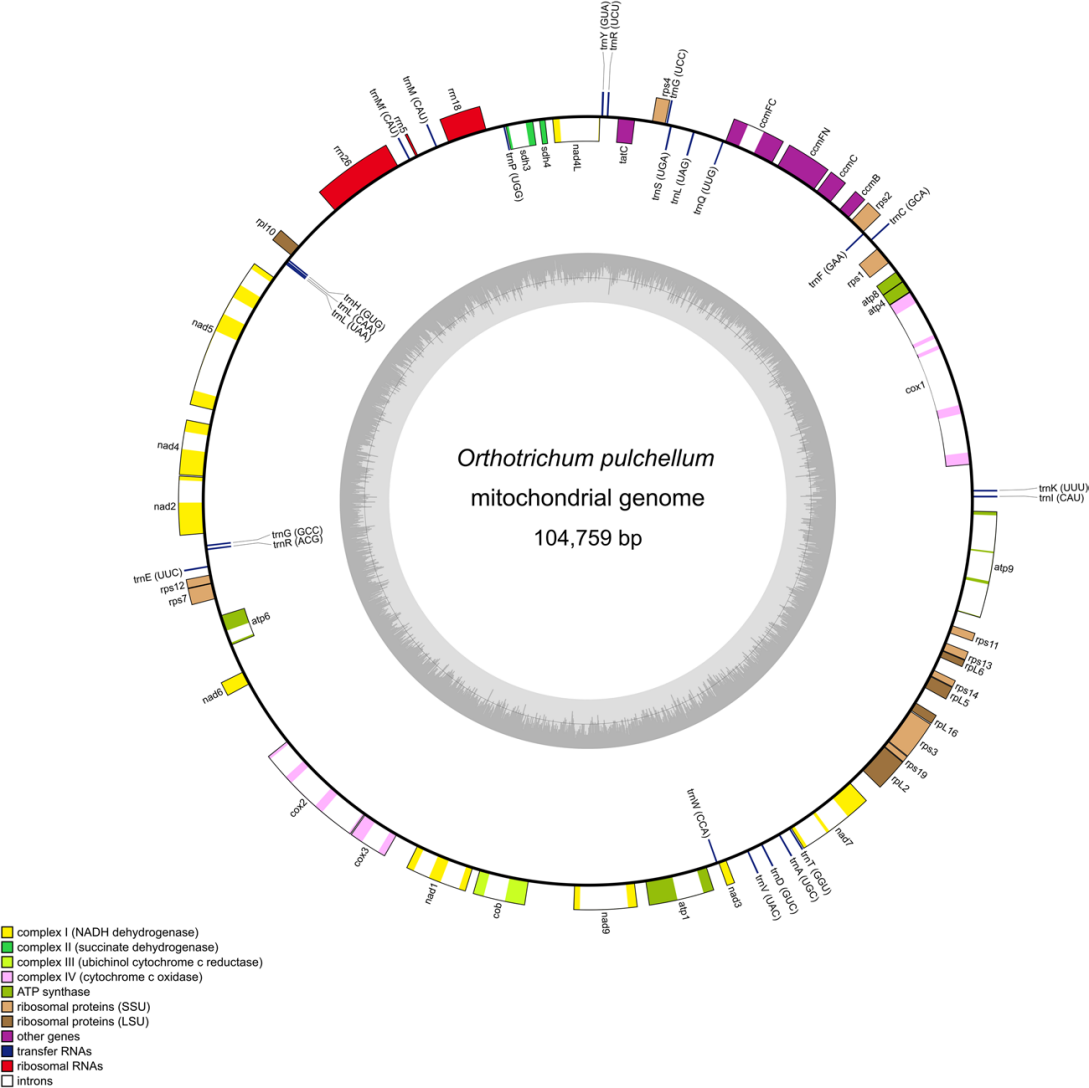
Gene map of the mitogenome of *Orthotrichum pulchellum*. Genes inside and outside the outer circle are transcribed in counterclockwise and clockwise directions, respectively. The genes are color coded based on their function. The inner circle visualizes the G/C content.

The developed libraries were sequenced to produce approximately 3–6 million paired-end reads, of which 6–10% were mapped to the mitogenome (see Supplementary Table S1). The sizes of the obtained mitogenomes ranged from 104,351 bp in *Stoneobryum mirum* D.H. Norris & H. Rob. to 104,785 bp in *Orthotrichum callistomum* Bruch & Schimp. (see Supplementary Table S1). The newly sequenced mitogenomes contained 40 protein-coding genes, three ribosomal RNA (rRNA) sequences, and 24 transfer RNA (tRNA) sequences. The order and localization of these genes are illustrated in Fig. 1.

### Orthotricheae phylogenetic reconstruction and mitogenomic evolution

**Figure 2:**
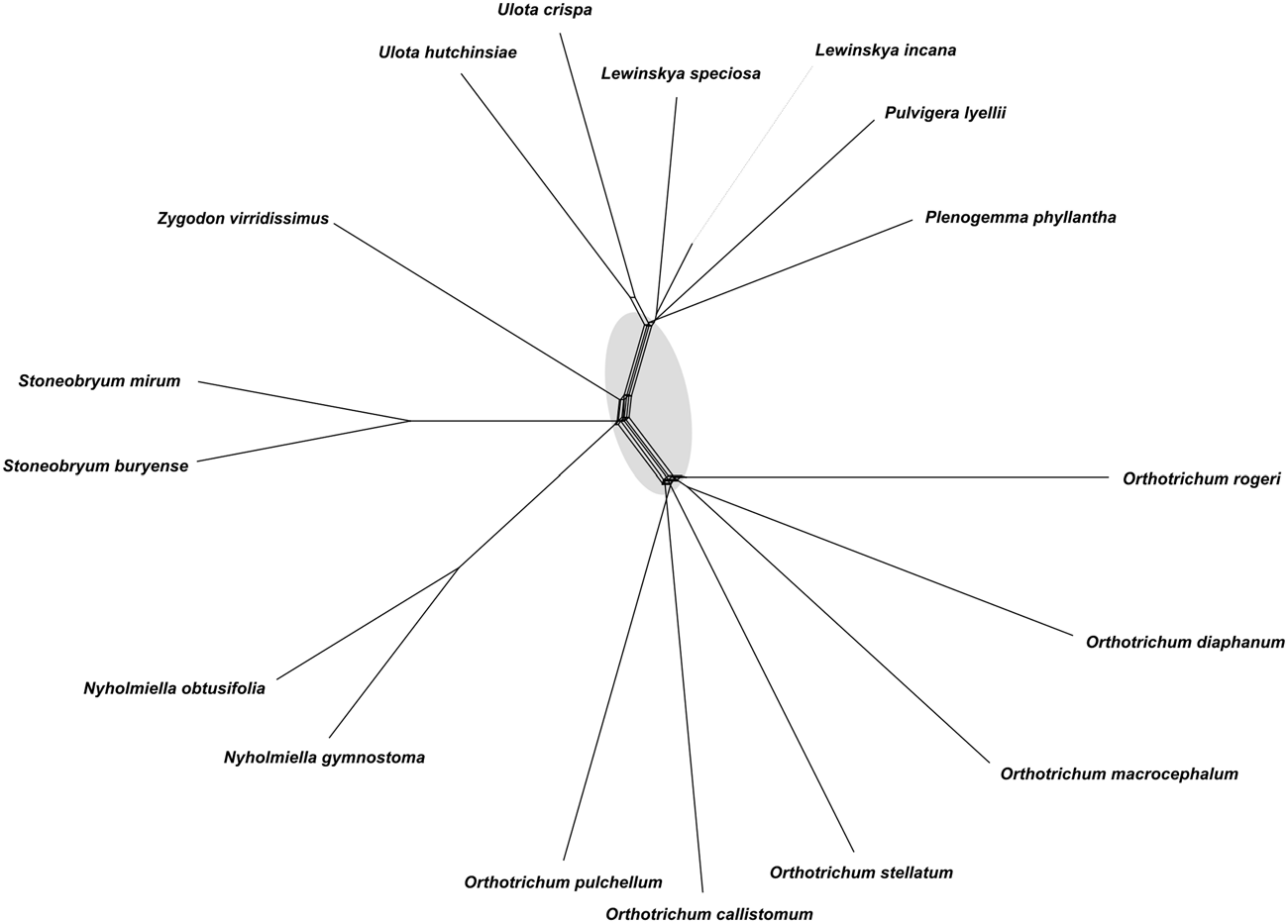
A supernetwork of 64 of 92 Orthotrichaceae mitochondrial single-locus trees constructed using SplitsTree. Parallelograms indicate incongruence among single-locus trees, and gray-shaded areas encompass the competing splits among major Orthotrichaceae lineages.

Alignments of 92 regions extracted from complete mitogenomes were used to construct phylogenetic trees using the ML method. Among them, 64 resolved at least one significantly supported (85% or more bootstrap support) clade, and those trees were used as input trees for SplitsTree. The super network analysis resolved all genera of the Orthotricheae as monophyletic (Fig. 2). Single-region trees differed in the relationships among these genera, but the lack of direct parallelism between OTUs suggested a lack of genes that could be transferred via hybridization and introgression.

**Figure 3:**
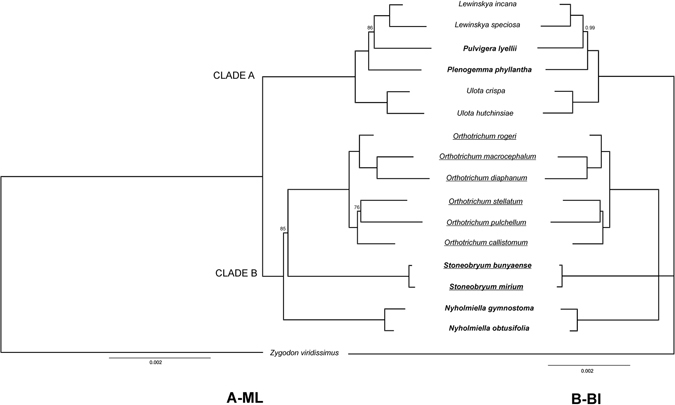
The phylogenetic relationships of 17 Orthotrichaceae species based on complete mitogenome sequences: A - Maximum Likelihood (ML), B - Bayesian Interference (BI). The bootstrap values (ML) and posterior probabilities for BI trees lower than the maximum values (100 and 1.0, respectively) are shown at the nodes. The underlined species have cryptoporous stomata, and taxa in bold are dioecious.

Phylogenetic reconstruction based on the ML and BI methods produced trees with similar topologies (Fig. 3), and two well-supported clades were identified in all analyses. The first clade (A) comprised *Ulota* species and large taxa of the genus *Orthotrichum* s.l. with phaneroporous stomata, namely, *Lewinskya* and *Pulvigera* ^20, 21^. Dioecious *Plenogemma phyllantha* (Brid.) Plášek, Sawicki & Ochyra did not form a monophyletic clade with the monoecious species, and all phylogenetic analyses suggested that this taxon should be considered as sister to *Pulvigera* and *Lewinskya* species (maximum BS and BI support). The monoecious *Lewinskya incana* (Müll. Hal.) F. Lara, Garilleti & Goffinet and *L*. *speciosa* (Nees) F. Lara, Garilleti & Goffinet formed a monophyletic, well-supported clade (0.99 PP and 86% BS) with dioecious *Pulvigera lyellii* (Hook. & Taylor) Plášek, Sawicki & Ochyra. The second main clade (B) comprised the genera *Orthotrichum* s.s., *Nyholmiella* and *Stoneobryum*, but the phylogenetic relationships among them were only partially resolved. The ML and BI analyses differed in the support of the *Stoneobryum* clade as sister to *Orthotrichum* s.s. In the case of the ML tree, *Stoneobryum* was resolved as sister to *Orthotrichum* s.s., with significant (85.2%) bootstrap support indicating *Nyholmiella* as a basal genus for clade B (Fig. 3A). However, the BI did not support this topology, leaving the *Nyholmiella*-*Stoneobryum* relationship unresolved (Fig. 3B). Both analyses were congruent in resolving relationships among species of *Orthotrichum* s.s., where both clades had maximum BS and BI support. The first clade contained *O*. *rogeri* Brid., which was resolved as sister to *O*. *diaphanum* Brid. and *O*. *macrocephalum* F. Lara, Garilleti & Mazimpaka. The second clade of *Orthotrichum* s.s. contained *O*. *callistomum*, which was resolved as sister to *O*. *pulchellum* Brunt. and *O*. *stellatum* Brid.

The analysis of the complete mitochondrial genome clarified numerous doubts concerning the evolution of taxa from clade B, which included cryptopore-containing taxa from *Nyholmiella*, *Stoneobryum*, and the small monoecious *Orthotrichum* s.s. genus. The results of this phylogenomic analysis indicated that the *Nyholmiella*, represented by two species (*N*. *obtusifolia* (Brid.) Holmen & Warncke and *N*. *gymnostoma* (Brid.) Holmen & Warncke), is sister to the monoecious species of *Orthotrichum* s.l. with immersed stomata, including *O*. *pulchellum*, *O*. *rogeri*, *O*. *stellatum*, and *O*. *callistomum* (which represents the separate subgenus *Callistoma*).

The phylogenetic analyses of partitioned data sets (CDS, 20 most variable CDS, non-coding regions and 20 most variable non-coding regions) resulted in poorly resolved trees without any incongruences with trees based on the complete mitogenomes (see Supplementary Figs SF1–4).

### Hotspots of variation within non-coding regions

**Figure 4:**
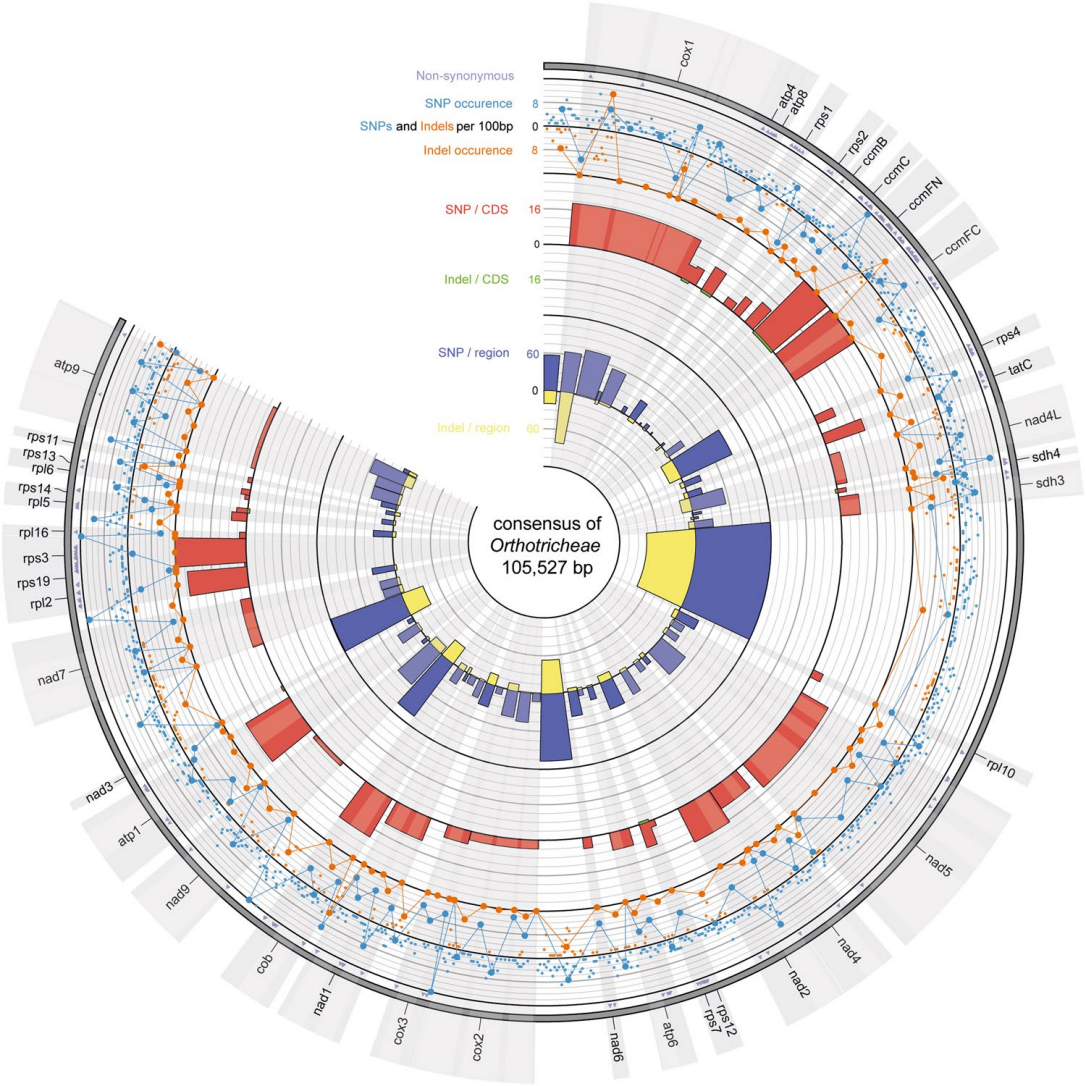
The pattern of the SNP/indel variation of analyzed Orthotricheae mitogenomes.

A total of 174 regions were identified in the mitogenomes of the species analyzed, including 90 coding regions (CDSs, tRNAs, and rRNAs), 27 introns, and 57 spacers. Among the non-coding regions longer than 100 bp (for bias elimination), the highest number of polymorphic sites (8.06%) occurred in intron 4 of the *cox*1 gene. In this 1798 bp-long fragment, 64 single nucleotide polymorphisms (SNPs) and 81 indels were identified (Fig. 4, see Supplementary Table S2). The intergenic spacer between the *cob* and *nad*9 genes (1,982 bp) was the second most variable mitogenome fragment, with 6.81% of polymorphic sites, including 104 SNPs and 31 indels (see Supplementary Table S2). Only slightly lower variation (6.58% of polymorphic sites) was detected in the more-than-five-times-shorter (365 bp) *nad*1-*cob* spacer, including 14 SNPs and 10 indels. Among introns, the highest variation was found in the introns of the *cox*1 gene in the aforementioned intron 4 and intron 2. The latter included 15 SNPs and six indels (5.29% of polymorphic sites). The third most variable intron was the 1556-bp-long intron 3 of *atp*9 (4.63% of polymorphic sites), which included 58 SNPs and 14 indels. The highest number of mutations (227 SNPs and 77 indels) was found in the *sdh*3-*rpl*10 spacer, which was the longest intergenic spacer in the analyzed mitogenomes (10,761 bp). The variation of all identified intergenic spacers is shown in Supplementary Table S2.

### Hotspots of variation within coding regions

Mutations (427 SNPs and six indels) were detected in 40 of 90 coding regions (see Supplementary Table S2), and single 3-bp-long indels were found in *atp*4, *rps*1, *ccm*FN, *sdh*4, *rps*7, and *rps*14. The most variable genes in the tribe Orthotricheae were c*cm*FC, which is responsible for cytochrome c biogenesis, and *tat*C, which encodes a TatC protein essential for the correct membrane translocation mechanism (38 and 19 SNPs, respectively). The remaining genes involved in cytochrome c biogenesis were less variable, including nine SNPs found in *ccm*B (one nonsynonymous) and 12 SNPs found in *ccm*C (eight nonsynonymous). By contrast, the *ccm*FN gene, with 2.11% of polymorphic sites, was fifth among the most variable coding regions (26 of 39 SNPs were nonsynonymous), after the *sdh*3 (nine SNPs, five nonsynonymous) and *rps*3 (35 SNPs, 19 nonsynonymous) genes. More than 10 SNPs were detected in 15 genes. The variability of those genes and others not discussed here is presented in Supplementary Table S2.

The dN/dS was also calculated for each gene, and ratios >1 were detected in the following genes: *ccm*FC, *ccm*FN, *rps*1, *rpl*2, *tat*C, *cob* and *nad*1. Several genes (*nad*3, *cox*2, *rps*12, *nad*4 L, *nad*7, *rps*19, *rpl*6, and *rps*11) only exhibited synonymous mutations, but these genes were also characterized by a low number of SNPs (1–5 SNPs per gene).

## Discussion

The complete sequences of mitogenomes confirmed previous hypotheses that postulated the structural stability of moss genomes^27, 30^. The gene content and order of the *Lewinskya*, *Nyholmiella*, *Orthotrichum*, *Plenogemma*, *Pulvigera*, *Stoneobryum*, *Ulota*, and *Zygodon* Hook. & Taylor mitogenomes (Fig. 1) were identical to those in previously researched Orthotrichaceae species^27, 31, 32^. Phylogenetic analyses based on complete mitogenomes were partially congruent with those of previous studies. For instance, the results of previous molecular studies^17–19^ indicated that *Nyholmiella* was a sister group to *Orthotrichum* species with immersed stomata. *Orthotrichum callistomum*, which belongs to the separate monospecific subg. *Callistoma* (according to Lewinsky)^14^, was not analyzed in previous studies; however, based on its diagnostic character, which is an unusual configuration of the endostome^14, 15^, *O*. *callistomum* is the most distinct species within *Orthotrichum*. This result was, however, not significantly reflected in the infrageneric arrangement of *Orthotrichum* s.l. in other studies^33^.

The endostome segments of *O*. *callistomum* are joined above to form a ring; they are also heavily papillose and bear trabeculae on their inner surface. There is no other species from the *Orthotrichum* s.l. with such a combination of features. Therefore, Lewinsky^14^ also accepted this species as a single representative of one of seven subgenera of *Orthotrichum* s.l.

Although Podpěra^34^ included *O*. *callistomum* in sect. *Callistoma*, which was later validated by Iwatsuki and Sharp^35^, Lewinsky^33^ decided to elevate the section to subgenus as *O*. subg. *Callistoma* (Z.Iwats. & Sharp) Lewinsky. She argued that the unique morphological features of *O*. *callistomum* warranted its inclusion in its own subgenus instead of its inclusion in the section. Moreover, Lewinsky^33^ referred to a similar reflection by Vitt^36^ and used similar reasoning to propose the establishment of a separate subgenus, *O*. subg. *Exiguifolia* Vitt for *Orthotrichum exiguum* Sull., which was later moved to the genus *Leratia* Broth. & Paris based on phylogenetic analyses^17^. However, the results of the present study do not support the distinctiveness of this subgenus, because all of the phylogenetic analyses placed *O*. *callistomum* in a clade containing species belonging to *O*. subg. *Pulchella* (Schimp.) Vitt, effectively merging subg. *Callistoma* and subg. *Pulchella* into a single subgenus.

Regarding the position of the genus *Stoneobryum*, the phylogenies based on complete mitogenomes were partially incongruent with the results of a previous study^17^ that drew conclusions from evolutionary inferences based on four loci (two plastid, one nuclear 26S, and one mitochondrial *nad*5). Goffinet *et al*.^17^ resolved *Stoneobryum* and *Sehnemobryum* as sister clades to the *Nyholmiella* and *Orthotrichum* s.s. The ML analysis based on complete mitogenomes placed *Stoneobryum* as sister to cryptoporous *Orthotrichum* species (Fig. 3A), with 85% bootstrap support, whereas the BI analysis left phylogenetic relationships between *Stoneobryum*, *Nyholmiella* and *Orthotrichum* unresolved (Fig. 3B). The inclusion of *Sehnemobryum* species in this analysis would likely help to resolve this incongruence. Unfortunately, the mitogenome assembly was not available, despite several attempts, and this was likely due to highly degraded organellar DNA.

The phylogenetic position of *Stoneobryum* based on ML analysis could be better explained by evolutionary features proposed by Lewinsky^14^, who suggested that a single evolutionary event leading to the presence of cryptoporous stomata from phaneroporous ancestry could be a fundamental dichotomy within *Orthotrichum* s.l. The results presented in this study are compatible with that hypothesis, grouping all cryptoporous taxa into a monophyletic clade (Fig. 3A). However, the position of *Stoneobryum* remained unresolved based on the BI analysis.

Regarding the appearance of cryptopores, the ML analysis of mitogenomes confirmed that cryptopores appeared only once during the evolution of *Orthotrichum* s.l. in an event that was independent of the change of the sexual reproduction system from dioecy toward monoecy. Moreover, cryptoporous taxa of *Orthotrichum* and *Stoneobryum* share a common ancestor with *Nyholmiella*. However, this scenario was not confirmed (or rejected) by BI analysis. Most likely, ongoing studies of the plastomes and large sets of nuclear genes will finally resolve this problem.

In agreement with previous studies^17–19^, the phylogenetic inferences based on the data from complete mitogenomes did not support the monophyly of dioecious species of the tribe Orthotricheae. However, the A and B clades distinguished differ with respect to the patterns of sexual system evolution. According to molecular phylogenies of mosses, sexual systems are often not correlated with phylogenetic relationships^36, 37^, and the evolutionary mechanisms associated with the shift between dioecy and monoecy could be as complex as sex determination mechanisms in plants^38, 39^. Based on the present and previous studies^16–19^, monoecy independently evolved from dioecy at least twice in clades A and B. For instance, the basal taxa in clade A are monoecious (*Ulota crispa* (Hedw.) Brid. and *U*. *hutchinsiae* (Sm.) Hamm.), but dioecious *Plenogemma phyllantha* and *Pulvigera lyellii* were resolved as basal taxa for *Lewinskya*. A simpler picture of the evolution of sexual systems in the tribe Orthotricheae is evident in clade B, where dioecious genera *Nyholmiella* and *Stoneobryum* were resolved as basal taxa, which suggests that the shift of sexual systems in this group only occurred once.

The results presented in this study are in agreement with those of previous studies based on single plastid regions as well as mitochondrial and nuclear genomes^17, 19, 40^ and support recent nomenclatural decisions^20, 21^. However, the results of these earlier studies indicated that the common clade containing *P*. *phyllantha* and *Lewinskya* species was not statistically supported. Only recent studies based on three plastid genes (*trn*G, *trn*L-F and *atp*B-*rbc*L) and incomplete ITS2 loci resolved a monophyletic *Ulota* clade, and only in two out of four analyses^41^.

Resolving phylogenetic inferences among the closely related Orthotrichaceae genera requires large data sets based on complete organellar genomes or transcriptomic data, and these analyses are good examples of how the ‘omics’ era could aid in the resolution of evolutionary questions in plant systematics. The phylogenetic tree of Orthotricheae based on the mitogenome confirms the previously observed polyphyletic nature of *Orthotrichum* s.l. and *Ulota* s.l., and this tree molecularly supports recent taxonomical rearrangement^20^. However, since the taxon sampling is limited (especially in the case of the genera *Lewinskya* and *Ulota*), the phylogenetic inferences presented in this study have a rather preliminary character.

Since the recent reporting of complete moss mitogenomes^27, 30, 32, 42, 43^, only scarce data concerning potential hotspots of variability have been presented^44^. The comparison of two *O*. *diaphanum* mitogenomes revealed only single nonsynonymous substitutions in the *rps*1 and *ccm*FN genes and noted the latter as a potential hotspot in the Orthotrichaceae^44^, which also confirms the results presented above. Comparative mitogenomics between *Physcomitrella patens* (Hedw.) Bruch & Schimp. and *Marchantia polymorpha* L. revealed that *nad*1, *nad*5, and *cox*3 were the most polymorphic genes, and that *rpl*2 had the highest number of nonsynonymous mutations^39^. In our study, these genes were characterized by moderate variation (see Supplementary Table S2), but *rpl*2 was in the top three most-variable genes based on the number of nonsynonymous mutations (see Supplementary Table S2). The results also support earlier studies that reported different evolutionary patterns associated with mitochondrial gene evolution in mosses and vascular plants^45^. The most variable Orthotricheae genes that were responsible for the biogenesis of cytochrome c (*ccm*FC and *ccm*FN) are among the most conserved genes in vascular plant mitogenomes, especially in monocots^45^. However, species with *ccm*FC pseudogenes are also known to exist^46^. In the genus *Silene* L., *atp*1 was found to be the most variable among mitochondrial genes^47^, and it was also one of the most polymorphic genes in the present study.

The intergenic and intronic hotspots of the Orthotricheae identified in the present study do not correspond to those described in vascular plant mitogenomes. The phylogenetically informative and quickly evolving intron 4 of the *nad*5 gene in the genus *Abies* Mill^48^. does not exist in the known moss mitogenomes. However, most of the intergenic variation in vascular plant mitogenomes is related to repeats (≥50 bp) that are present in genomes in many copies^49^, which possibly lead to non-homologous, intragenomic recombination^50–52^. The lack of these repeats in moss mitogenomes is considered the main cause of their structural stability^27^.

**Figure 5:**
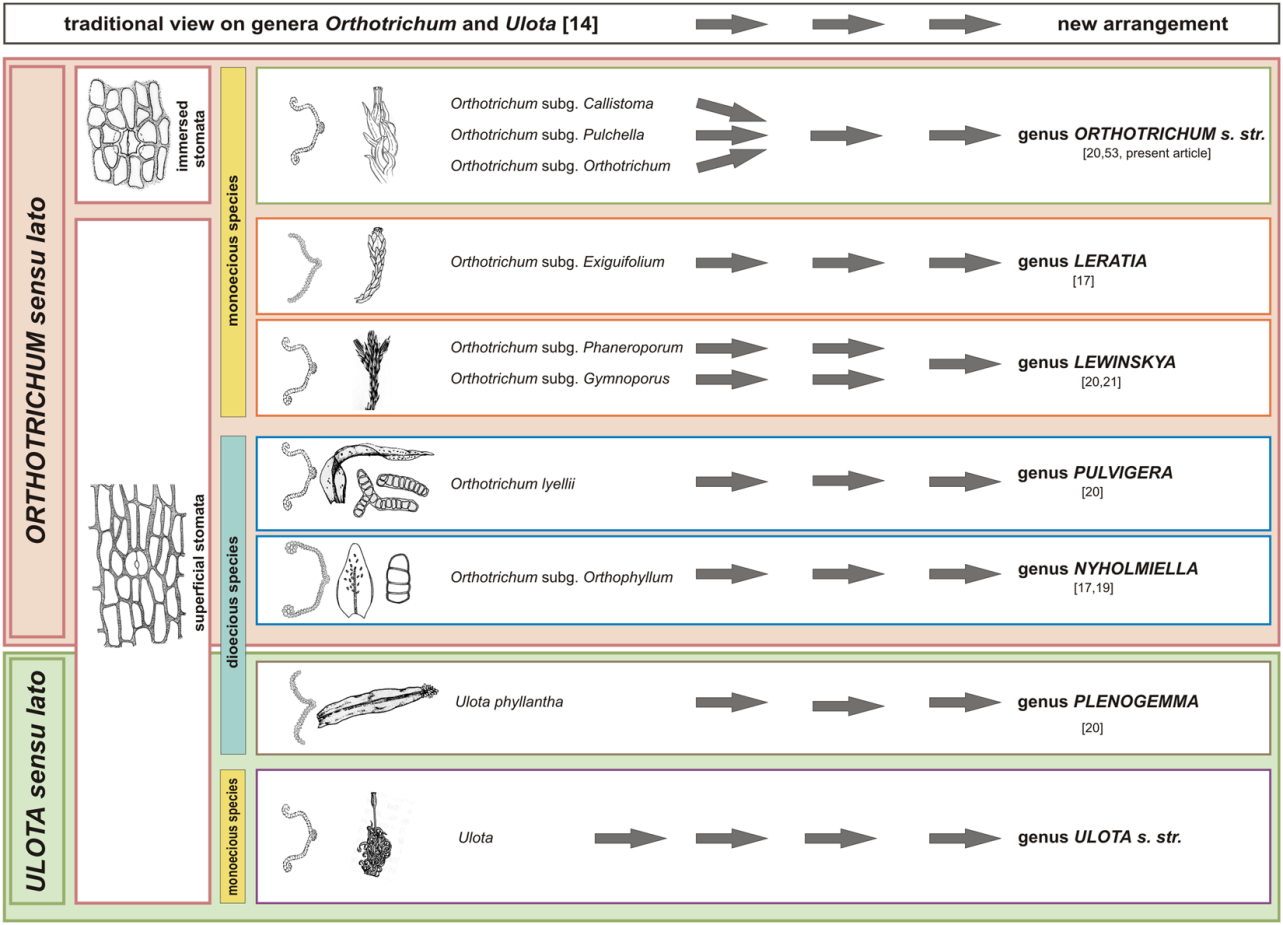
Taxonomic changes within the traditionally conceived genera *Orthotrichum* and *Ulota*. The diagram shows how the historical conception (left side) based on Lewinsky^14^ was changed into the new arrangement^20^ (right side).

A new taxonomical arrangement of the traditionally conceived genera *Orthotrichum* s.l. and *Ulota* s.l. was presented by Plášek *et al*.^20^. Taxonomic changes are graphically expressed in Fig. 5. A critical feature associated with the reclassification of this taxonomic group is the type and position of stomata. For instance, *Orthotrichum* s.s. is the only genus with cryptoporous stomata. Among the genera with phaneroporous stomata, an important classification character is the ploidy level. Monoecious taxa include the *Ulota* s.s. and *Lewinskya* genera, and the genera share superficial stomata and recurved leaf margins. However, *Ulota* species can be easily distinguished from *Lewinskya* by the presence of quadrate to rectangular hyaline cells that form a marginal border at the leaf base. Moreover, brood bodies are never produced in *Ulota* species, and asexual reproduction by propagules within *Lewinskya* species is extremely rare. Therefore, the dioecious genera of the Orthotricheae can be mainly characterized by the production of abundant gemmae, and this apparently compensates for the rare incidence of sexual reproduction in comparison to the monoecious species. The dioecious group consists of *Nyholmiella*, *Stoneobryum*, *Pulvigera*, and *Plenogemma*, and the latter two taxa have been recognized as new genera^20^. *Stoneobryum*, whose phylogenetic position remains ambiguous, is the only dioecious genus with cryptoporous stomata. *Nyholmiella* differs from *Pulvigera* and *Plenogemma* primarily based on the presence of an ovate leaf with an obtuse apex and somewhat incurved leaf margins, and the latter two genera have leaves that are linear-lanceolate or lanceolate with an acute to acuminate apex. While the gemmae in *Pulvigera* are scattered more or less equally on the adaxial leaf surface, in *Plenogemma* the conspicuous clusters of fusiform brownish gemmae are situated on the protruding costa of the upper leaves.

## Materials and Methods

### Material

Genomic libraries were created with the use of DNA isolated in our previous work^18, 19, 53^, with the exceptions of DNA samples from *Stoneobryum bunyaense* D.H. Norris & H. Rob., *S*. *mirum*, *Lewinskya incana*, and *Orthotrichum callistomum*, which were isolated using the Zymo Plant/Seed DNA kit (Zymo Research Corp., Irvine, CA, USA) following the manufacturer’s instructions. Specimen details and corresponding GenBank accession numbers are listed in Supplementary Table S1. Based on previous results^16–19^, *Zygodon* was selected as the outgroup. The DNA quantity was estimated with the use of a Qubit fluorimeter system and Quant-IT ds-DNA BR Assay kit (Invitrogen, Carlsbad, NM, USA).

Genomic libraries for MiSeq sequencing were developed using the Nextera XT Kit (Illumina, San Diego, CA, USA) and 1 ng of DNA; however, the normalization procedure was conducted using the Kapa Library Quantification Kit for Illumina (Kapa, Wilmington, MA, USA). The numbers of created and sequenced genomic libraries for each taxon are given in Supplementary Table S1.

The size distributions of the libraries were checked using primer sequences provided in the Sequencing Library qPCR Quantification Guide (Illumina). PCRs were performed in 20-µL reaction mixtures containing 3 µL of the genomic library, each primer at 1.0 µM, 1.5 mM MgCl2, 200 µL of dNTPs (dATP, dGTP, dCTP, and dTTP), 1X PCR buffer, and 1 U of OpenEx Taq polymerase (OpenExome, Warsaw, Poland). PCRs were performed under the following thermal conditions: (1) initial denaturation, 5 min at 94 °C; (2) denaturation, 30 s at 94 °C; (3) annealing, 30 s at 52 °C; (4) elongation, 1 min at 72 °C; and (5) final elongation, 7 min at 72 °C. Steps 2–4 were repeated 34 times. The PCR products were separated using a QIAxcel capillary electrophoresis system (Qiagen, Hilden, Germany). Electrophoresis was performed using a QIAxcel High Resolution Kit (Qiagen) with a 15–1,000-bp alignment marker and a 25–1,000-bp size marker; standard OL500 settings were used in the electrophoresis program. Validated libraries were pooled according to guidelines found in the Kapa Library Quantification Kit for Illumina. Genomic libraries were sequenced using MiSeq 500v2 and 600v3 cartridges, which supported the acquisition of 2 × 250- and 2 × 300-bp paired-end reads, respectively.

### Mitogenome assembly

The obtained reads were mapped to the previously published mitogenome of *Orthotrichum speciosum* ^31^ using Geneious R6 software (Biomatters, Auckland, New Zealand) with default medium-low sensitivity settings and the previously detailed assembly workflow^27^. Briefly, fragments located between gaps and overlapping but incongruent sequences were extracted and moved to separate files. Raw paired-end reads with 100 iterations were mapped to every fragment using customized settings (minimum sequence overlap of 150 and 99% overlap identity). The resulting contigs underwent *de novo* assembly using the default settings in Geneious, and all available reads were mapped to the draft assembly of the mitogenome to determine coverage and to check for incongruence between the mapped sequences. Annotated mitogenome GenBank files were used to draw gene maps using the OrganellarGenome DRAW tool^53^, and the maps were examined for further comparisons of gene order and content.

### Phylogenetic and variation analyses

Eleven mitogenomes obtained in the present study and six previously published genomes^25, 27, 31, 44^ were aligned using the Mauve genome aligner^54^, and two regions containing tandem repeats were adjusted manually. Unambiguously aligned DNA sequences were used in the phylogenetic analyses.

The consistency among single genes and intergenic regions was evaluated by the maximum-likelihood (ML) analysis for every locus that contained parsimony informative characters using the RAxML v7.2.3 plugin^55^ for Geneious 7.0.3 with the GTR + I + G model and bootstraps estimated using 500 replicates.

Potential conflicts among genes and spacers were visualized by constructing a supernetwork of 62 trees using SplitsTree4^56^ according to the approach given by Liu *et al*.^57^, except the number of replicates was increased to 500 and the software used for creating consensus tree was Geneious 7.0.3.

PartitionFinder2^58^ was used to determine the best partitioning schemes and corresponding nucleotide substitution models. The data-set blocks were predefined a priori based on protein-coding genes (CDS), tRNA and rRNA, introns and intergenic spacers as well as for first, second and third position for each of CDS. The Bayesian information criterion (BIC) and the ‘greedy’ algorithm with branch lengths estimated as unlinked were used to search for the best-fit scheme. The partitions with optimal substitution models are given in Supplementary Table S3. Phylogenetic reconstruction was also conducted using four partitioned data sets, which included coding regions, the 20 most variable protein-coding genes, intergenic spacers (including introns) and the 20 most variable non-coding regions (see Supplementary Figs SF1–4).

Phylogenetic analyses were conducted using ML and BI methods. The ML analysis was performed using RAxML version 8.2.4^55^ with the partitioning scheme and nucleotide substitution blocks generated by PartitionFinder2, using default parameters. Bootstrap analyses were performed with 2,000 replicates to assess nodal support.

Bayesian analysis (BI) was conducted using MrBayes 3.2^59^, and the MCMC algorithm was run for 20,000,000 generations (sampling every 1,000) with four incrementally heated chains (starting from random trees). The visual inspection of Tracer 1.3^60^ plots was used to examine the parameters and to determine the number of generations needed to reach stationarity, which occurred at approximately 300,000 generations. Therefore, the first 500 trees were discarded as burn-in, and the remaining trees were used to develop a Bayesian consensus tree.

All regions, including coding regions, introns, and intergenic spacers, were extracted to identify divergent regions within the 16 mitogenomes (excluding the *Zygodon* outgroup) for additional phylogenetic applications. The percentage of variable characters within each region was calculated, and the number of nucleotide substitutions and indels (potentially informative characters (PIC)) among the 13 mitogenomes was determined for each region. Indels were scored in this study because they tend to be prevalent and phylogenetically informative^61, 62^. The preliminarily performed phylogenetic analyses based on the data set excluding indels resulted in trees with identical topology as presented here but, in some cases, with lower bootstrap support. Indels and nucleotide substitutions within indels were scored as independent, single characters to obtain the results comparable with those of previous studies^45, 48^. The percentage of variation (P) in each region (exons, introns, and spacers) was described using the formula of O’Donnell^63^, which was successfully used to identify variation in plastid regions^64, 65^. Mitogenome variation was visualized using Circos^66^ and a custom Python script.

To identify mitochondrial loci undergoing adaptation, dN/dS ratios were calculated for each gene. This measure quantifies selection pressures by comparing the rate of synonymous substitutions (dS), which are presumed neutral, to the rate of nonsynonymous substitutions (dN), which possibly experience selection^67^. A codon-based test of neutrality was conducted using the Nei-Gojobori method^68^ as implemented in MEGA7 software^69^, with default settings.

To estimate the level of substitution saturation, we used the approach of Xia *et al*.^70^ as implemented in DAMBE5 software^71^. The amount of substitutional saturation was calculated for four scenarios: the first codon position, the second codon position, the combined the first and the second codon positions, and the third codon position. Since no significant saturation was detected, all three partitions involving codon positions were used in the phylogenetic analyses.

## Conclusions

The phylogenetic analyses based on complete mitogenome sequences support the new taxonomic classification within *Orthotrichum* s.l. (Fig. 5) as proposed by Plášek *et al*.^20^. The thirteen newly sequenced mitogenomes confirmed their stable gene order and structure in the mosses. Given the limited data availability, it is difficult to propose any universal pattern of mitochondrial gene variation in mosses. However, several ongoing studies of mitogenome variation patterns among different moss families will likely provide additional background information related to this subject.

## Electronic supplementary material

Supplementary materials
