# Supplementary material for: Mitogenomic analyses support the recent division of the genus Orthotrichum (Orthotrichaceae, Bryophyta)
Source: Sci Rep. 2017 Jun 30;7:4408. doi: 10.1038/s41598-017-04833-z (PMC5493672; doi:10.1038/s41598-017-04833-z)
Supplement: Supplementary file 1 — Supplementary materials [file 41598_2017_4833_MOESM1_ESM.pdf]

Mitogenomic analyses support the recent division of the genus Orthotrichum (Orthotrichaceae, Bryophyta)

Jakub Sawicki, Vítězslav Plášek, Ryszard Ochyra, Monika Szczecińska, Monika Ślipiko, Kamil Myszczyński and Tomasz Kulik

Supplementary Table S1. Sample species, voucher specimens and sequencing results of species used in this study. The newly sequenced specimens are bolded.

Species	voucher	sequencing results	mitogenome lenght	mitogenome coverage	GenBank accession number
Lewinskya incana (Orthotrichum incanum)	**El Rosario, Chile, NYBG 1010874**	**2,643,409 2x300bp pair-end reads**	**104,736**	**57x**	**KX578028**
Lewinskya speciosa (Orthotrichum speciosum)	Sawicki et al. 2014		104,747	87x	KM288416
Nyholmiella gymnostoma	**Notre Dame Bay, Canada, NYBG 151512**	**3,077,215 2x300bp pair-end reads**	**104,638**	**69x**	**KX578030**
Nyholmiella obtusifolia	**Olsztyn, Poland, OL-M 10109**	**6,234,126 2x250bp pair-end reads**	**104,603**	**105x**	**KX702279**
Orthotrichum callistomum	**Yulong Xue Shan, China, KUN B0012532**	**2,618,194 2x300bp pair-end reads**	**104,785**	**58x**	**KX578029**
Orthotrichum diaphanum	Vigalondo et al. 2016		104,756	106x	KT373970
Orthotrichum macrocephalum	Vigalondo et al. 2016		104,624	60x	KT823696
Orthotrichum rogeri	Sawicki et al. 2015		104,634	61x	KM873610
Orthotrichum pulchellum	**Olloy, Belgium, OP186523**	**3,002,982 2x250bp pair-end reads**	**104,759**	**68x**	**KR297063**
Orthotrichum stellatum	Liu et al. 2014		104,131	unknown	KC784958
Pulvigera lyellii (Orthotrichum lyellii)	**Orlovice, Czech Republic, TC8912**	**3,208,238 2x300 bp pair-end reads**	**104,704**	**77x**	**KR297064**
Plenogemma phyllantha (Ulota phyllantha)	**Lærdalsøyri, Norway, OSTR B493**	**3,924,686 2x250bp pair-end reads**	**104,671**	**72x**	**KX578033**
Stoneobryum bunyaense	**Kiangaro Mts, Australia, US 00070599**	**3,668,008 2x300 bp pair-end reads**	**104,352**	**59x**	**KX578031**
Stoneobryum mirium	**South Africa, Amatola Mountains, Katberg, DUKE 0016791**	**9,601,004 2x300 bp pair-end reads**	**104,345**	**130x**	**KY620883**
Ulota crispa	**Blansko, Czech republic, OP OP187011**	**4,340,122 2x250bp pair-end reads**	**104,636**	**67x**	**KX578032**
Ulota hutchinsiae	Liu et al. 2014		104,608	unknown	KC784952
Zygodon viridissimus	**Couvin, Belgium, OP186541**	**2,998,756 2x250bp pair-end reads**	**103,098**	**48x**	**KX711975**

Supplementary Table S2. Mutations found in the alignment (excluding outgroup) i coding and non-coding regions of the thirteen mitogenomes.

**Coding Regions**							**Spacers and Introns**					
**Gene**	Fragment length (bp)	SNP	Indel	All	Non-synonymous	% of variation	**Region**	Fragment length (bp)	SNP	Indel	All	% of variation
*ccm*FC	1,455	38	0	38	27	2.61	*cox*1 intron 4	1,798	64	81	145	8.06
*tat*C	735	19	0	19	10	2.59	*cob/nad*9	1,982	104	31	135	6.81
*sdh*3	396	9	0	9	5	2.27	*nad*1/*cob*	365	14	10	24	6.58
*rps*3	1,617	35	0	35	19	2.16	*ccm*FN/*ccm*FC	270	7	10	17	6.30
ccmFN	1,896	39	1	40	26	2.11	*nad*6/*cox*2	2,982	107	53	160	5.37
*rps*14	300	5	1	6	3	2.00	*cox*1 intron 2	397	15	6	21	5.29
*rpl*2	1,392	27	0	27	16	1.94	*cox*2/*cox*3	19	1	0	1	5.26
*sdh*4	261	4	1	5	3	1.92	*nad*7/*rpl*2	748	36	3	39	5.21
*rps*7	693	11	1	12	7	1.73	*nad*4L/*sdh*4	302	14	1	15	4.97
*ccm*B	528	9	0	9	1	1.70	*nad*3/*nad*7	3,144	119	36	155	4.93
*nad*4L	303	5	0	5	0	1.65	*cox*1/*atp*9	2,029	67	33	100	4.93
cob	1,221	20	0	20	9	1.64	*rps*13/*rps*11	473	15	8	23	4.86
*rps*1	810	12	1	13	6	1.60	*rpl*16/*rpl*5	749	31	5	36	4.81
*ccm*C	759	12	0	12	8	1.58	*rps*3/*rpl*16	21	1	0	1	4.76
*rps*4	588	9	0	9	6	1.53	*ccm*C/*ccm*FN	129	5	1	6	4.65
*atp*8	525	8	0	8	3	1.52	*atp*9 intron 3	1,556	58	14	72	4.63
*atp*1	1,557	23	0	23	7	1.48	*rps*4/*tat*C	735	28	6	34	4.63
*atp*4	552	7	1	8	3	1.45	*nad*2 intron 1	979	40	4	44	4.49
*rpl*5	561	7	0	7	3	1.25	*sdh*4/*sdh*3	278	8	4	12	4.32
*cox*1	1,569	19	0	19	2	1.21	*cox*3/*nad*1	1,231	36	17	53	4.31
*nad*2	1,470	17	0	17	3	1.16	*sdh*3 intron 1	910	30	9	39	4.29
*nad*1	987	11	0	11	6	1.11	*ccm*FC intron 1	780	30	3	33	4.23
*rps*13	369	4	0	4	2	1.08	*rps*11/*atp*9	646	25	2	27	4.18
*atp*6	759	8	0	8	5	1.05	*rps*1/*rps*2	946	33	5	38	4.02
*rpl*10	540	5	0	5	2	0.93	*nad*4L intron 1	1,775	55	16	71	4.00
*atp*9	225	2	0	2	2	0.89	*nad*1 intron 1	752	28	2	30	3.99
*nad*6	606	5	0	5	2	0.83	*ccm*FC/*rps*4	2,736	89	20	109	3.98
*rps*12	381	3	0	3	0	0.79	*nad*9 intron 1	2046	68	13	81	3.96
*cox*3	798	6	0	6	3	0.75	*atp*9 intron 2	1,196	43	3	46	3.85
*rpl*16	408	3	0	3	1	0.74	*cox*2 intron 3	1,438	42	11	53	3.69
*nad*5	2,031	14	0	14	5	0.69	*tat*C/*nad*4L	788	23	6	29	3.68
*nad*4	1,488	10	0	10	1	0.67	*rps*14/*rpl*6	606	19	3	22	3.63
*rpl*6	306	2	0	2	0	0.65	*cox*1 intron 1	1,638	56	3	59	3.60
*rps*11	357	2	0	2	0	0.56	*atp*6/*nad*6	1,250	40	5	45	3.60
*rps*2	726	4	0	4	2	0.55	*cob* intron 1	947	25	9	34	3.59
*cox*2	762	4	0	4	0	0.52	*rpl*10/*nad*5	1,190	35	7	42	3.53
*nad*7	1,182	5	0	5	0	0.42	*cox*2 intron 2	1,406	47	1	48	3.41
*rps*19	282	1	0	1	0	0.35	*atp*9 intron 1	1,417	43	4	47	3.32
*nad*9	588	2	0	2	2	0.34	*atp*1 intron 1	1,157	35	3	38	3.28
*nad*3	357	1	0	1	0	0.28	*nad*5/*nad*4	623	14	6	20	3.21
							*nad*2/*rps*12	1,779	45	11	56	3.15

							*nad*7 intron 2	1,021	28	4	32	3.13
							*rps*2/*ccm*B	161	4	1	5	3.11
							*nad*7 intron 1	1,032	27	5	32	3.10
							*rps*12/*atp*6	746	21	2	23	3.08
							*nad*5 intron 2	840	21	4	25	2.98
							*sdh*3/*rpl*10	10,761	227	77	304	2.83
							*nad*9/*atp*1	466	10	3	13	2.79
							*cox*1 intron 3	2,648	72	1	73	2.76
							*nad*5 intron 1	852	20	2	22	2.58
							*nad*4 intron 1	781	17	3	20	2.56
							*atp*B/*rps*1	483	11	1	12	2.48
							*ccm*B/*ccm*C	506	9	2	11	2.17
							*atp*6 intron 1	618	10	3	13	2.10
							*nad*5 intron 3	2,632	49	0	49	1.86
							*atp*1/*nad*3	539	10	0	10	1.86
							*nad*1 intron 2	859	14	1	15	1.75
							*cox*2 intron 1	1,028	13	1	14	1.36
							*cox*3 intron 1	831	11	0	11	1.32
							*nad*4/*nad*2	26	0	0	0	0.00
							*rpl*2/*rps*19	3	0	0	0	0.00
							*rps*19/*rps*3	6	0	0	0	0.00
							*rpl*5/*rps*14	4	0	0	0	0.00
							*rpl*6/*rps*13	3	0	0	0	0.00

Supplementary Table S3. The partitions with optimal substitution models generated by the PartitionFinder2.

**Subset**	**Best Model**	**Number of sites**	**Partition names**
1	GTR+I+G	879	atp6_intron_1, start_trnI-CAU
2	GTR	2225	nad4_exon_2_pos2, nad5_exon_2_pos1, nad2_exon_1_pos1, cob_exon_2_pos1, trnR-UCU, trnG-UCC, trnW-CCA, trnI-CAU, trnY-GUA_nad4L, trnK-UUU, nad7_exon_2_pos2, trnY-GUA, trnV-UAC, trnA-UGC, trnE-UUC, nad7_exon_3_pos2, cox2_exon_3_pos3, nad9_exon_2_pos3, cox3_exon_2_pos2, atp9_exon_3_pos3
3	GTR	1525	trnI-CAU_trnK-UUU, rpL16_CDS_pos3, rpl10_CDS_pos3, rps12_CDS_pos3, atp1_exon_1_pos3, rps14_CDS_pos3, ccmFC_exon_1_pos3, atp1_exon_2_pos2
4	GTR+G	5099	rps1_trnC-GCA, trnI-CAU_cox1, tatC_trnR-UCU, trnA-UGC_trnT-GGU, cob_nad9, trnV-UAC_trnD-GUC
5	GTR	1521	tatC_CDS_pos2, sdh4_CDS_pos2, sdh3_exon_2_pos2, cox1_exon_5_pos1, sdh3_exon_1_pos2, trnG-UCC_rps4, sdh4_CDS_pos1, atp6_exon_1_pos3, cox1_exon_1_pos2, tatC_CDS_pos3, cox1_exon_3_pos1, rps4_CDS_pos1, nad4L_exon_1_pos2
6	GTR+I+G	1541	rps12_CDS_pos1, rps13_CDS_pos1, cox1_exon_1_pos3, atp6_exon_1_pos1, rps7_CDS_pos1, nad9_exon_2_pos1, cox1_exon_3_pos2, rpL6_CDS_pos1, cox1_exon_5_pos2, rpL16_CDS_pos1, rpL16_CDS_pos2, rps12_CDS_pos2, cox2_exon_1_pos1
7	GTR	927	cox1_exon_5_pos3, trnL-UAG, trnS-UGA, trnR-ACG, trnP-UGG, rps11_CDS_pos2, cox1_exon_2_pos3, rps19_CDS_pos2, rrn5_rRNA, trnQ-UUG
8	GTR+I+G	1798	cox1_intron_4
9	GTR	528	atp9_exon_1_pos3, atp6_exon_2_pos2, nad4L_exon_1_pos3, nad4_nad2, cox1_exon_2_pos2, rps3_rpl16, cox1_exon_4_pos1
10	GTR	380	cox1_exon_4_pos2, nad4L_exon_1_pos1, cox2_exon_4_pos1, atp1_exon_2_pos1

11	GTR	616	tatC_CDS_pos1, cox1_exon_4_pos3, sdh3_exon_1_pos3, ccmC_ccmFN, sdh3_exon_2_pos1
12	GTR+I+G	2648	cox1_intron_3
13	GTR+I+G	1787	atp6_exon_2_pos3, cox1_exon_1_pos1, rpl10_CDS_pos1, cox1_exon_3_pos3, cox2_exon_3_pos1, nad7_exon_3_pos3, atp8_CDS_pos1, trnH-GUG, atp1_exon_1_pos2, cox2_exon_1_pos2, trnL-UAA
14	GTR	1213	rps4_tatC, cox1_intron_2, trnL-CAA
15	GTR+I+G	1788	nad5_exon_4_pos3, nad1_exon_2_pos1, nad5_exon_2_pos3, nad3_CDS_pos3, ccmB_CDS_pos2, atp9_exon_4_pos1, nad5_exon_4_pos2, cox1_exon_2_pos1, nad1_exon_3_pos1, nad9_exon_2_pos2, cox2_exon_2_pos1, cox3_exon_1_pos1, nad2_exon_1_pos2, ccmFC_exon_2_pos1
16	GTR+G	4768	sdh3_intron_1, atp4_CDS_pos2, nad4L_intron_1, cox1_intron_1, ccmFN_ccmFC
17	GTR	1385	trnF-GAA, rps14_CDS_pos1, ccmC_CDS_pos1, nad4_exon_1_pos1, rps2_CDS_pos2, nad7_exon_1_pos2, nad7_exon_2_pos3, atp4_CDS_pos1, rpL5_CDS_pos2, rps11_CDS_pos3
18	GTR	919	rps2_ccmB, nad4_exon_2_pos1, trnH-GUG_trnL-CAA, rpL6_CDS_pos3, atp4_CDS_pos3, atp9_exon_2_pos3, nad9_exon_1_pos2
19	GTR+I+G	1980	ccmFN_CDS_pos3, rpl2_CDS_pos3, rps3_CDS_pos3, atp8_CDS_pos3, atp8_CDS_pos2
20	GTR+I+G	2206	atp6_nad6, atpB_rps1, rps13_rps11
21	GTR+G	1205	nad5_exon_3_pos3, nad4_exon_1_pos3, ccmC_CDS_pos3, rps1_CDS_pos1, ccmFC_exon_1_pos2, rps19_rps3
22	GTR	900	rps1_CDS_pos2, ccmB_CDS_pos1, rpl10_CDS_pos2, ccmFC_exon_2_pos2, nad9_exon_1_pos1
23	GTR	1458	nad5_exon_2_pos2, cox3_exon_2_pos3, rps1_CDS_pos3, atp9_exon_1_pos2, cob_exon_1_pos2, cob_exon_2_pos2, ccmC_CDS_pos2, cox3_exon_2_pos1, nad1_exon_2_pos3
24	GTR	1011	rps2_CDS_pos1, trnG-GCC_trnR-ACG, nad5_exon_4_pos1, rps13_CDS_pos2, rpL6_CDS_pos2, cox2_exon_2_pos2, trnC-GCA, rps19_CDS_pos1
25	GTR+I+G	3156	atp1_trnW-CCA, trnC-GCA_trnF-GAA, atp9_end, rrn18_trnM-CAU, ccmFC_exon_1_pos1, nad3_trnV-UAC, cob_exon_1_pos1
26	GTR+G	132	trnF-GAA_rps2, trnS-UGA_trnG-UCC
27	GTR	716	nad7_exon_2_pos1, nad7_exon_1_pos3, nad9_exon_1_pos3, nad4L_exon_2_pos3, rps4_CDS_pos3, rps13_CDS_pos3, rps2_CDS_pos3
28	GTR	1324	nad7_exon_3_pos1, nad6_CDS_pos3, nad2_exon_2_pos3, ccmB_CDS_pos3, nad5_exon_1_pos3, cox2_exon_3_pos2
29	GTR+I+G	5927	nad9_atp1, sdh4_CDS_pos3, ccmFN_CDS_pos2, trnL-UAG_trnS-UGA, sdh4_sdh3, ccmFC_exon_2_pos3, ccmFN_CDS_pos1, cob_intron_1, cox3_nad1, ccmB_ccmC
30	GTR+I+G	3316	ccmFC_intron_1, nad9_intron_1, nad2_trnG-GCC
31	GTR	51	ccmFC_trnQ-UUG
32	GTR	2657	rpl10_trnH-GUG, trnMf-CAU_rrn26, rps11_atp9, trnQ-UUG_trnL-UAG
33	GTR+I+G	582	atp6_exon_2_pos1, sdh3_exon_2_pos3, rps4_CDS_pos2, cox2_exon_4_pos2, trnW-CCA_nad3
34	GTR+I+G	827	nad4L_sdh4, trnR-UCU_trnY-GUA, nad1_cob
35	GTR+I+G	7018	atp9_intron_3, cox2_intron_2, rps7_CDS_pos3, nad5_nad4, atp9_intron_1, rps3_CDS_pos1, rrn5_trnMf-CAU, nad7_intron_2, nad4L_exon_2_pos1
36	GTR	4539	nad4_intron_1, trnD-GUC, cox3_exon_1_pos2, trnG-GCC, nad5_intron_1, nad1_intron_2, rrn18_rRNA, nad4L_exon_2_pos2
37	GTR+I+G	482	nad3_CDS_pos1, atp9_exon_4_pos2, nad5_exon_1_pos1, atp6_exon_1_pos2, sdh3_exon_1_pos1, nad1_exon_2_pos2, cox2_exon_4_pos3
38	GTR	658	nad1_exon_3_pos3, nad1_exon_1_pos2, sdh3_trnP_UGG, nad4_exon_1_pos2, atp9_exon_4_pos3, nad2_exon_1_pos3, nad6_CDS_pos2
39	GTR+I+G	3366	trnP-UGG_rrn18, trnE-UUC_rps12, rrn26_rpl10
40	GTR	2573	trnM-CAU, rps14_CDS_pos2, trnMf-CAU, rpl2_CDS_pos2, cox2_intron_1, cox3_intron_1
41	GTR+I+G	3905	trnM-CAU_rrn5, nad6_cox2
42	GTR	3604	rrn26_rRNA, nad1_exon_1_pos1, rps11_CDS_pos1
43	GTR+I+G	1625	trnR-ACG_trnE-UUC, rps7_CDS_pos2, trnL-UAA_nad5
44	GTR	198	atp9_exon_3_pos2, atp9_exon_2_pos2, nad5_exon_1_pos2, nad1_exon_3_pos2
45	GTR	1568	nad5_intron_2, rpL5_CDS_pos1, rps3_CDS_pos2
46	GTR	874	nad6_CDS_pos1, nad5_exon_3_pos1, nad2_exon_2_pos1

47	GTR+I+G	1392	cox2_exon_2_pos3, nad3_CDS_pos2, nad2_exon_2_pos2, nad4_exon_2_pos3, nad5_exon_3_pos2, cox3_exon_1_pos3, atp9_exon_3_pos1
48	GTR	2632	nad5_intron_3
49	GTR	1731	nad2_intron_1, nad1_intron_1
50	GTR+I+G	1352	rps7_atp6, rps14_rpl6
51	GTR+I+G	413	rpL5_CDS_pos3, rps19_CDS_pos3, cox2_exon_1_pos3, nad1_exon_1_pos3
52	GTR+I+G	5412	atp9_intron_2, atp1_intron_1, cox2_intron_3, rpl2_CDS_pos1, atp1_exon_2_pos3, nad7_intron_1
53	GTR+I+G	407	cob_exon_2_pos3, cob_exon_1_pos3
54	GTR	469	atp9_exon_2_pos1, atp1_exon_1_pos1, atp9_exon_1_pos1, nad7_exon_1_pos1
55	GTR+G	2314	nad7_rpl2, trnD-GUC_trnA-UGC, rpl16_rpl5

Supplementary Figures SF1-4. The phylogenetic relationships of 17 Orthotrichaceae species based on partitioned mitogenomic datasets: SF1-AllCDS – dataset based on all protein-coding sequences, SF1-20CDS dataset based on 20 most variable sequenced; SF3-AllNC – dataset based on all non-coding regions; SF4-20NC – dataset based on the 20 most variable non-coding regions. The bootstrap values (ML) are given at nodes. The nodes with underlined bootstrap values are not supported by BI analyses (posterior probabilities lower than 0.95).

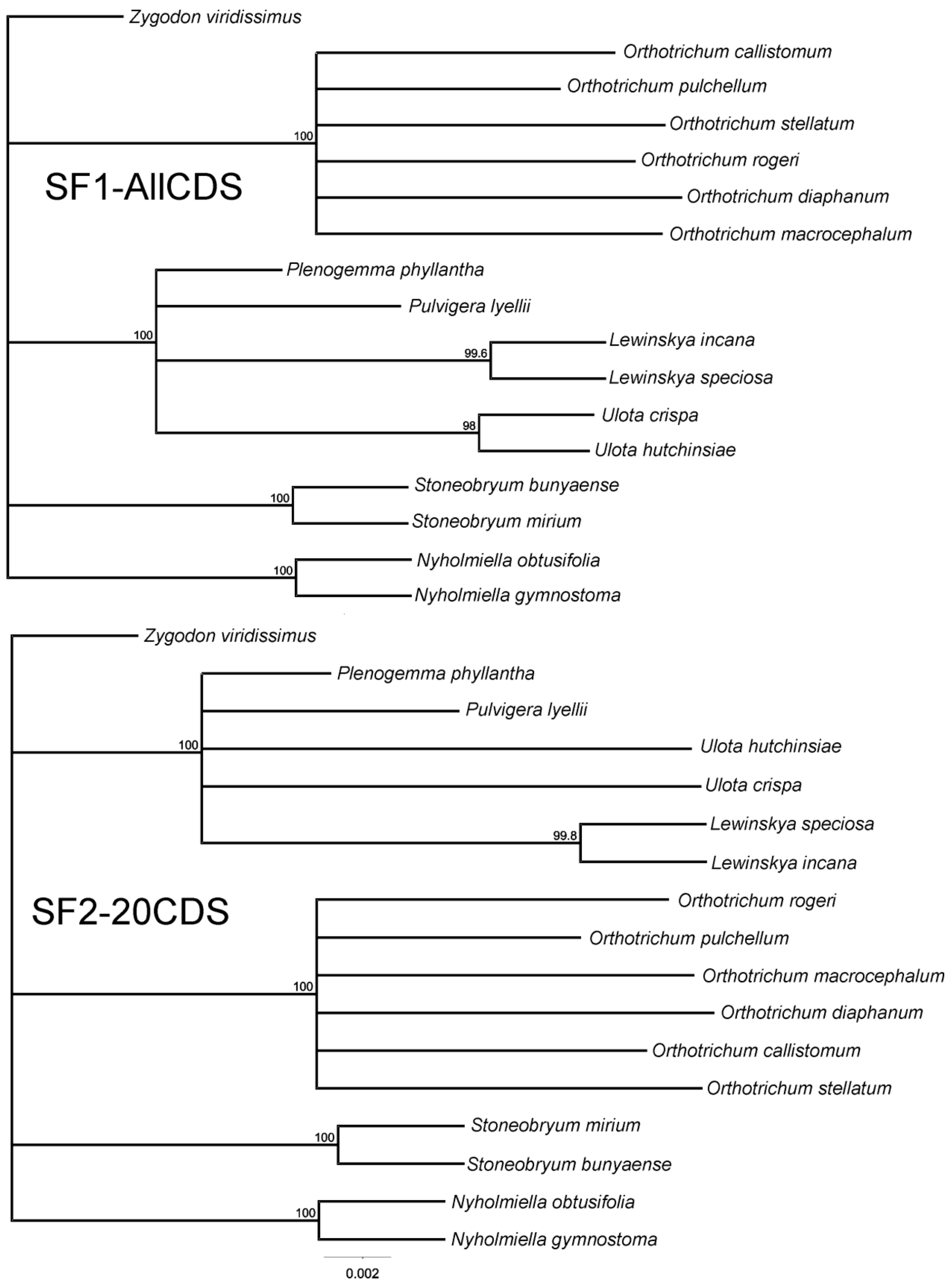

SF1-AllCDS
Zygodon viridissimus
Orthotrichum callistomum
Orthotrichum pulchellum
Orthotrichum stellatum
Orthotrichum rogeri
Orthotrichum diaphanum
Orthotrichum macrocephalum
Plenogemma phyllantha
Pulvigera lyellii
Lewinskya incana
Lewinskya speciosa
Ulota crispa
Ulota hutchinsiae
Stoneobryum bunyaense
Stoneobryum mirium
Nyholmiella obtusifolia
Nyholmiella gymnostoma
100
100
99.6
98
100
100
SF2-20CDS
Zygodon viridissimus
Plenogemma phyllantha
Pulvigera lyellii
Ulota hutchinsiae
Ulota crispa
Lewinskya speciosa
Lewinskya incana
Orthotrichum rogeri
Orthotrichum pulchellum
Orthotrichum macrocephalum
Orthotrichum diaphanum
Orthotrichum callistomum
Orthotrichum stellatum
Stoneobryum mirium
Stoneobryum bunyaense
Nyholmiella obtusifolia
Nyholmiella gymnostoma
100
99.8
100
100
100
0.002

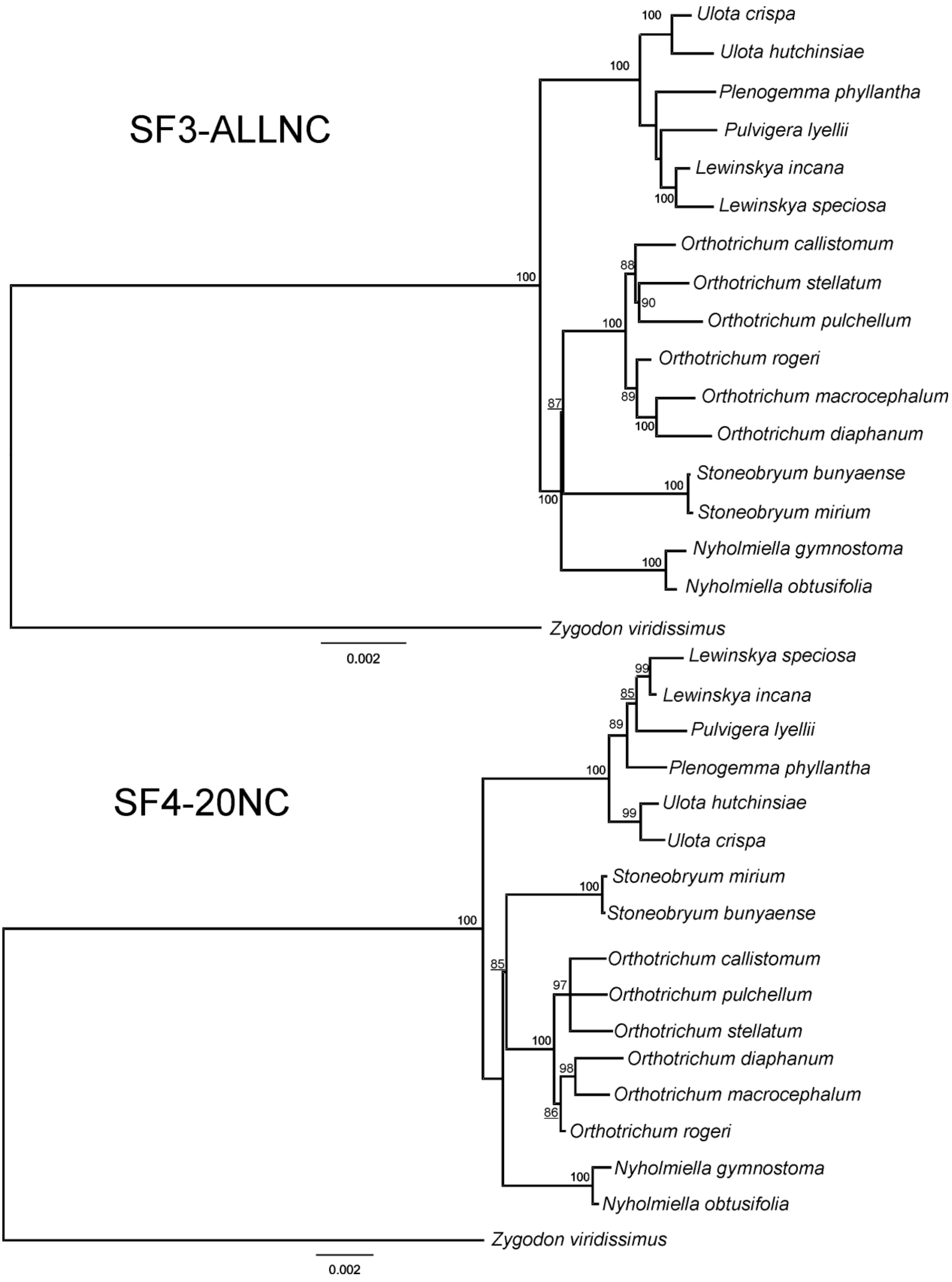
SF3-ALLNC
Ulota crispa
Ulota hutchinsiae
Plenogemma phyllantha
Pulvigera lyellii
Lewinskya incana
Lewinskya speciosa
Orthotrichum callistomum
Orthotrichum stellatum
Orthotrichum pulchellum
Orthotrichum rogeri
Orthotrichum macrocephalum
Orthotrichum diaphanum
Stoneobryum bunyaense
Stoneobryum mirium
Nyholmiella gymnostoma
Nyholmiella obtusifolia
Zygodon viridissimus
0.002
SF4-20NC
Lewinskya speciosa
Lewinskya incana
Pulvigera lyellii
Plenogemma phyllantha
Ulota hutchinsiae
Ulota crispa
Stoneobryum mirium
Stoneobryum bunyaense
Orthotrichum callistomum
Orthotrichum pulchellum
Orthotrichum stellatum
Orthotrichum diaphanum
Orthotrichum macrocephalum
Orthotrichum rogeri
Nyholmiella gymnostoma
Nyholmiella obtusifolia
Zygodon viridissimus
0.002
